# The epidemiology of dystonia: the Hannover epidemiology study

**DOI:** 10.1007/s00415-022-11310-9

**Published:** 2022-08-11

**Authors:** Dirk Dressler, Eckart Altenmüller, Ralf Giess, Joachim K. Krauss, Fereshte Adib Saberi

**Affiliations:** 1 Movement Disorders Section, Department of Neurology, Hannover Medical School, Carl-Neuberg-Str. 1, 30625 Hannover, Germany; 2 Institute of Music Physiology and Musicians’ Medicine, Hannover University of Music, Drama and Media, Hannover, Germany; 3 Hildesheim, Germany; 4 Department of Neurosurgery, Hannover Medical School, Hannover, Germany; 5 IAB - Interdisciplinary Working Group for Movement Disorders, Hamburg, Germany

**Keywords:** Dystonia, Epidemiology, Prevalence, Cervical dystonia, Blepharospasm, Writer’s cramp, Tardive dystonia, Musician’s dystonia, Psychogenic dystonia, Functional dystonia, Generalised dystonia, Spasmodic dysphonia, Segmental dystonia, Arm dystonia, Oromandibular dystonia, Botulinum toxin therapy

## Abstract

The prevalence of dystonia has been studied since the 1980s. Due to different methodologies and due to varying degrees of awareness, resulting figures have been extremely different. We wanted to determine the prevalence of dystonia according to its current definition, using quality-approved registries and based on its relevance for patients, their therapy and the health care system. We applied a service-based chart review design with the City of Hannover as reference area and a population of 525,731. Barrier-free comprehensive dystonia treatment in few highly specialised centres for the last 30 years should have generated maximal dystonia awareness, a minimum of unreported cases and a high degree of data homogeneity. Prevalence [*n*/1mio] and relative frequency is 601.1 (100%) for all forms of dystonia, 251.1 (42%) for cervical dystonia, 87.5 (15%) for blepharospasm, 55.2 (9%) for writer’s cramp, 38.0 (6%) for tardive dystonia, 32.3 (5%) for musician’s dystonia, 28.5 (5%) for psychogenic dystonia, 26.6 (4%) for generalised dystonia, 24.7 (4%) for spasmodic dysphonia, 20.9 (3%) for segmental dystonia, 15.2 (3%) for arm dystonia and 13.3 (2%) for oromandibular dystonia. Leg dystonia, hemidystonia and complex regional pain syndrome-associated dystonia are very rare. Compared to previous meta-analytical data, primary or isolated dystonia is 3.3 times more frequent in our study. When all forms of dystonia including psychogenic, generalised, tardive and other symptomatic dystonias are considered, our dystonia prevalence is 3.7 times higher than believed before. The real prevalence is likely to be even higher. Having based our study on treatment necessity, our data will allow better allocation of resources for comprehensive dystonia treatment.

## Introduction

Dystonia was defined in 1984 by the Ad Hoc Committee of the Dystonia Medical Research Foundation (DMRF) [9] as the occurrence of sustained involuntary muscle contractions causing twisting, repetitive movements and abnormal postures.

The current concept of dystonia was developed in the mid-1980s by C David Marsden and Stanley Fahn [8] when they—for the first time—unified various hitherto independent conditions under the term dystonia. Dystonia covers a large number of different manifestations occurring with a wide spectrum of severities caused by various aetiologies and probably reflecting numerous different underlying pathophysiologies. It is still defined entirely by its clinical phenomenology. Due to its inhomogeneity, various classifications have been proposed.

The relatively recent introduction of the dystonia concept still causes a continued awareness deficit generating large numbers of undiagnosed patients and complicating the interpretation of older clinical data. Older clinical data may also be impaired by outdated classification systems. Lack of technical parameters may generate diagnostic uncertainty, at least for the non-expert. Dystonia’s wide spectrum of manifestations still generates terminological problems complicating data base searches. It also causes the separation of dystonia patients into various medical specialties. Its considerable variability of severities produces large number of mildly affected patients not pursuing contact to the medical system.

All this makes epidemiological studies and their interpretation challenging. We wanted to study the prevalence of dystonia based on current definitions and classifications and with a novel service-based approach to generate data, which are relevant for patients, their therapy and the health care system.

## Methods

### Design

The study is a service-based chart review to determine the point prevalence of dystonia patients requesting and/or requiring therapy in the City of Hannover on December 31st, 2014.

### Patients

Patient inclusion criteria consisted of (1) diagnosis of dystonia (2) request of the patient and/or requirement as decided by the physician to undergo treatment (3) registered address within the City of Hannover. There was no exclusion of specific dystonia forms, such as psychogenic dystonia, tardive dystonia, axial dystonia, genetic dystonia etc.

### Database

Patients were identified from the general patient database of the Department of Neurology and the Botulinum Toxin Therapy Registry of the Movement Disorders Section of the Department of Neurology of Hannover Medical School. Databases were maintained and supervised by local movement disorders specialists. A certain proportion of patients were contributed by two co-authors (EA, RG), also specialised in movement disorders and particularly dystonia.

### Dystonia population

Dystonia was diagnosed according to the above mentioned DMRF criteria. According to current concepts, psychogenic dystonia was classified as dystonia, rather than pseudo-dystonia. Functional dystonia is a synonym of psychogenic dystonia. Nocturnal oromandibular dystonia, i.e., bruxism, was included in this study. Its relationship to oromandibular dystonia will be discussed below. Psychogenic dystonia was diagnosed by physical examination and anamnestic features as previously suggested [10]. Tardive dystonia was diagnosed, when dystonia occurred under or up to 1 year after exposure to dopamine receptor blocking agent intake for at least 1 month. Dystonia as an additional symptom in widespread and diffuse brain damage, e.g., perinatal brain damage/infantile cerebral palsy or hypoxic brain damage, was not included in this study. Likewise, spasticity including elements of dystonia [5] was not included in this study. As camptocormia is not considered to be predominantly dystonic in origin, it was not included in this study. Axial dystonia, however, was considered to be dystonic.

Dystonia was classified according to the localisation of its main manifestation. The main dystonic manifestation was the manifestation for which treatment was requested and/or offered. Additional localisations were considered for classification only, when they were treated. The following localisations were considered: Cervical dystonia (CD): involvement of neck and shoulder muscles. Blepharospasm (BS): involvement of periocular muscles. Oromandibular dystonia (OMD): involvement of perioral, mandibular and lingual muscles. Spasmodic dysphonia (SD): involvement of laryngeal muscles. Additional involvement of pharyngeal muscles may be present. Arm dystonia (AD): involvement of arm muscles with spontaneous occurrence. Leg dystonia (LD): involvement of leg muscles. Segmental dystonia (SGD): involvement of two or more contiguous body regions. Hemidystonia (HD): exclusive involvement of muscles in one side of the body. Generalised dystonia (GD): involvement of muscles in more than one non-contiguous body regions regardless of their severity. In addition, patients may be classified according to pathophysiology with task-specific dystonia in the forms of writer’s cramp (WC) and musician's dystonia (MD). Other task-specific dystonia such as athlete’s cramps would have been included, if they would have occurred. Aetiology may also have been used for classification. In tardive dystonia (TRD), dystonia was caused by exposure to dopamine receptor blocking agents, in psychogenic dystonia (PSY), it was caused by a psychological mechanism. TRD and PSY patients were classified under their aetiologies rather than under their dystonia localisation. In complex regional pain syndrome-associated dystonia (CRPD), a complex regional pain syndrome was believed to have caused dystonia.

### Reference area

The reference area is the City of Hannover. This reference area was chosen because of its long-established and comprehensive dystonia infrastructure generating a high degree of dystonia awareness, its barrier-free access to treatment generating a minimum of unreported cases and few and well interconnected treatment providers generating a high degree of data homogeneity.

### Dystonia treatment in the reference area

Dystonia treatment in the reference area is provided by five specialised and interconnected treatment centres.

The Movement Disorders Section of the Department of Neurology at Hannover Medical School (TC-DD) was started in 1991 by Professor R Dengler. It was one of the first centres in Germany providing botulinum toxin therapy. In 2008, one of the authors (DD) was appointed the first professor for movement disorders in Germany. In this capacity, he was able to restructure and expand BT operations in Hannover considerably. He communicated pro-actively with the regional referring neurologists. He organised teaching sessions on dystonia and BT therapy for referring neurologists, general practitioners and physiotherapists. He connected with patient organisations and generated an active media presence. The centre’s primary catchment area is the whole North of Germany with many patients also coming from other parts of Germany and the whole of Europe and the Middle East.

The Department of Neurosurgery of Hannover Medical School (TC-JKK) directed by Professor JK Krauss operates an internationally renowned centre for Deep Brain Stimulation and other movement disorders surgery.

The Department of Phoniatry of Hanover Medical School (TC-MP) is run by Professor M Ptok. He is one of the leading phoniatricians providing BT therapy in Germany.

The Institute of Music Physiology and Musicians’ Medicine at Hannover University of Music, Drama and Media (TC-EA), directed by Professor E Altenmüller, holds the world’s largest groups of patients with musician’s dystonia. He directly collaborated in this project and provided anonymised patient data.

One private neurologist (Niedergelassener Neurologe), Doctor R Giess (TC-RG), also sees dystonia patients frequently and applies BT therapy. He is serving patients from all state and private health insurances. He also collaborated in this project and provided anonymised patient data.

Dystonia therapy is offered at the Movement Disorders Section according to internationally recognised standards [4]. The main dystonia treatment is BT therapy. It is offered for all dystonia indications except PSY. With an annual BT consumption in excess of 20,000 standard vials of onabotulinumtoxinA, abobotulinumtoxinA, incobotulinumtoxinA and rimabotulinumtoxinB the Movement Disorders Section is one the busiest BT centres in Europe. Dystonia forms without official registrations in Germany are covered by off-label therapy. Except for a nominal prescription fee, BT therapy is free of charge for all patients. Other dystonia therapies provided, include drug treatments, physiotherapy, occupational therapy, psychotherapy and re-training programs for musician’s dystonia. Deep brain stimulation is performed by TC-JKK frequently in combination with BT therapy. Treatment of MD is performed by TC-EA, treatment of SD by TC-MP.

### Reference population

As provided by the Census Office of the City of Hannover and shown in Table 1, the population of the reference area on December 31st 2014 was 525,731 with registered addresses spread over 29 postal areas. The population in the reference area consists of German nationals and non-German inhabitants. The ethnic background of the population is diverse. Depending on the definitions applied, at least half of the Hannover population is considered of having a ‘migratory background’, i.e., having a foreign nationality alone or in combination with a German citizenship. About one third of them coming from the European Union and the rest mainly from various Islamic countries (official website of the City of Hannover). The patient’s nationality and ethnic background was not monitored in this study, nor was there a way to identify the ethnic composition in the different postal areas.

**Table 1:** Raw data

PA	P [*n*]	TN [*n*]	CD [*n*]	BS [*n*]	WC [*n*]	OMD [*n*]	MD [*n*]	SD [*n*]	GD [*n*]	SGD [*n*]	AD [*n*]	HD [*n*]	LD [*n*]	PSY [*n*]	TRD [*n*]	CRPD [*n*]
30,159	8865	5	5													
30,161	25,426	27	9		7	1	2	2		2				2	2	
30,163	23,476	14	4	3	3		1		2	1						
30,165	23,498	5	2	2										1		
30,167	19,161	6	3		1	1		1								
30,169	9909	4		3				1								
30,171	16,784	15	4	4	1		4	1							1	
30,173	19,223	6	3								1			1	1	
30,175	7080	10	3		1	1	5									
30,177	14,300	5	2	2	1											
30,179	16,126	10	5	4			1									
30,419	43,076	18	6	3	1	1			1	1	1			3	1	
30,449	20,431	9	3	1					1		2		1	1		
30,451	16,575	4	2	1						1						
30,453	19,947	13	6	3					1		1			1	1	
30,455	22,084	14	6	3	1				1			1		1	1	
30,457	19,401	9	3	1	2					1			1	1		
30,459	22,779	17	7	2		1		1	1	1					4	
30,519	32,166	20	9	2	5		1			1	1			1		
30,521	19	0														
30,539	20,596	8	3	2					3							
30,559	22,095	20	7	1	2			1	2	1	1					
30,625	17,991	13	6	1	1			1	1						3	
30,627	16,072	22	13	4	1	1									3	
30,629	16,479	13	5		1			4			1			1	1	
30,655	19,478	14	6	2	1	1	1			1					2	
30,657	16,484	9	3	1			1	1		1				2		
30,659	16,200	11	7	1			1		1							1
30,916	10	0														
**total**	**525,731**	**316**	**132**	**46**	**29**	**7**	**17**	**13**	**14**	**11**	**8**	**1**	**2**	**15**	**20**	**1**

Prevalence of dystonia in the City of Hannover and its postal areas

*PA* postal area, *P* population, *TN* total number of dystonia patients, *CD* cervical dystonia, *BS* blepharospasm, *WC* writer's cramp, *OMD* oromandibular dystonia, *MD* musician’s dystonia, *SD* spasmodic dysphonia, *GD* generalised dystonia, *SGD* segmental dystonia, *AD* arm dystonia, *HD* hemidystonia, *LD* leg dystonia, *PSY* psychogenic dystonia, *TRD* tardive dystonia, *CRPD* CRPS-associated dystonia

### Population of Germany

Hannover prevalence data were extrapolated to indicate the number of dystonia patients in Germany. For this, Statistisches Bundesamt (Census Office of the Federal Republic of Germany) provided the figure of the population of the Federal Republic of Germany on December 31st 2014. It was 81,198,000.

### Reference date

The reference date was 31st, December 2014.

### Parameters

Study parameters include the point prevalence in the reference area on the reference date (prevalence), the patient’s name (name), the patient’s sex (sex), the patient’s age on reference date (age), the patient’s address on reference date (postal area), the patient’s diagnosis (diagnosis) and information about the application of BT therapy.

### Ethical approval

The study was performed under the regulations of the local ethical committee of Hannover Medical School.

## Results

### Data source

89% patient data of this study originated from TC-DD, 6% from TC-EA and 5% from TC-RG. Patients from TC-EA included 18 patients with MD and 3 patients with WC.

### Raw data

Table 1 shows the raw data acquired in this study. The prevalence in the postal areas range from 212.8 to 1412.4/1mio.

### Total prevalence

The epidemiological data obtained in this study are shown in Table 2. Altogether 316 dystonia patients were identified in the reference area on the reference date. This equals a point prevalence of 601.1/1mio. Extrapolation suggests that there should be 48,806 dystonia patients in Germany.

**Table 2:** Epidemiological data of the Hannover cohort

Dystonia form	Age [years/mean±SD]	Sex ratio [m:f]	Frequency [% of all dystonia patients]	Prevalence [*n*/1mio]	Extrapolated prevalence in Germany [*n*]
Cervical dystonia	63.8 ± 13.9	0.3	42	251.1	20,387
Blepharospasm	69.4 ± 12.5	0.4	15	87.5	7105
Writer's cramp	62.0 ± 16.6	0.5	9	55.2	4479
Tardive dystonia	67.0 ± 19.7	0.3	6	38.0	3089
Musician's dystonia	50.2 ± 14.4	1.8	5^a^	32.3^a^	2626^a^
Psychogenic dyst	47.2 ± 20.8	1.1	5	28.5	2317
Generalised dystonia	51.7 ± 14.8	0.3	4	26.6	2162
Spasmodic dysphonia	60.4 ± 17.5	0.4	3	24.7	2008
Segmental dystonia	62.6 ± 13.5	0.2	3	20.9	1699
Arm dystonia	70.5 ± 12.5	0.6	3	15.2	1236
Oromandibular dyst	46.7 ± 15.8	0.8	2	13.3	1081
Leg dystonia			1		
Hemidystonia			0		
**Total**	**62.4 ± 16.1**	**0.4**	**100**	**601.1**	**48,806**

^a^Explanation in Discussion

### CD prevalence

CD patients are 63.8 ± 13.9 years. Their male/female ratio is 0.3. 42% of all dystonia patients suffer from CD. It is by far the most common dystonia manifestation. Its prevalence is 251.1/1mio. Extrapolation suggests 20,387 CD patients in Germany. 94% of patients accepted BT therapy, 6% preferred other therapies or did not wish therapy.

### BS prevalence

BS patients are 69.4 ± 12.5 years. Their male/female ratio is 0.4. 15% of all dystonia patients suffer from BS. It is the second most common dystonia manifestation. Its prevalence is 87.5/1mio. Extrapolation suggests 7105 BS patients in Germany. 98% of patients accepted BT therapy, 2% preferred other therapies or did not wish therapy.

### WC prevalence

WC patients are 62.0 ± 16.6 years. Their male/female ratio is 0.5. 9% of all dystonia patients suffer from WC. It is the third most common dystonia manifestation. Its prevalence is 55.2/1mio. Extrapolation suggests 4479 WC patients in Germany. 59% of patients accepted BT therapy, 41% preferred other therapies or did not wish therapy.

### TRD prevalence

TRD patients are 67.0 ± 19.7 years. Their male/female ratio is 0.3. 6% of all dystonia patients suffer from TRD. It is the fourth most common dystonia manifestation. Its prevalence is 38.0/1mio. Extrapolation suggests 3089 TRD patients in Germany. 65% of patients accepted BT therapy, 35% preferred other therapies or did not wish therapy.

### MD prevalence

MD patients are 50.2 ± 14.4 years. Their male/female ratio is 1.8. 5% of all dystonia patients suffer from MD. With this, it would be the fifth most common dystonia manifestation. Its prevalence is 32.2/1mio. Extrapolation suggests 2626 MD patients in Germany. This seemingly high prevalence will be discussed below. All patients accepted trials of BT therapy.

### PSY prevalence

PSY patients are 47.2 ± 20.8 years. Their male/female ratio is 1.1. 5% of all dystonia patients suffer from PSY. It is the sixth most common dystonia manifestation. Its prevalence is 28.5/1mio. Extrapolation suggests 2317 PSY patients in Germany. Therapeutic recommendations were based on psychotherapy and physiotherapy, which were accepted by all patients. Antidystonic pharmacotherapy was not offered.

### GD prevalence

GD patients are 51.7 ± 14.8 years. Their male/female ratio is 0.3. 4% of all dystonia patients suffer from GD. It is the seventh most common dystonia manifestation. Its prevalence is 26.6/1mio. Extrapolation suggests 2162 GD patients in Germany. 64% of patients accepted BT therapy, alone or in combination with other therapies. 36% received other therapies usually including deep brain stimulation.

### SD prevalence

SD patients are 60.4 ± 17.5 years. Their male/female ratio is 0.4. 3% of all dystonia patients suffer from SD. It is the eighth most common dystonia manifestation. Its prevalence is 24.7/1mio. Extrapolation suggests 2008 SD patients in Germany. 85% of patients accepted BT therapy. 15% preferred other therapies, usually speech therapy, or did not receive any treatment.

### SGD prevalence

SGD patients are 62.6 ± 13.5 years. Their male/female ratio is 0.2. 3% of all dystonia patients suffer from SD. It is the ninth most common dystonia manifestation. Its prevalence is 20.9/1mio. Extrapolation suggests 1699 SGD patients in Germany. All patients accepted BT therapy.

### AD prevalence

AD patients are 70.5 ± 12.5 years. Their male/female ratio is 0.6. 3% of all dystonia patients suffer from SD. It is the tenth most common dystonia manifestation. Its prevalence is 15.2/1mio. Extrapolation suggests 1236 AD patients in Germany. 63% of patients accepted BT therapy, 37% preferred other therapies or chose not to be treated.

### OMD prevalence

OMD patients are 46.7 ± 15.8 years. Their male/female ratio is 0.8. 2% of all dystonia patients suffer from OMD. It is the eleventh most common dystonia manifestation. Its prevalence is 13.3/1mio. Extrapolation suggests 1081 OMD patients in Germany. All patients accepted BT therapy. Four of our OMD patients had nocturnal occurrence, only, three had continuous OMD. The age of the nocturnal OMD patients was 40.3 ± 7.3 years.

### LD prevalence

LD is extremely rare only occurring as a levodopa induced transient drug adverse effect in a patient with advanced idiopathic Parkinson’s disease.

### HD prevalence

HD is also extremely rare occurring in a single patient with contralateral basal ganglia damage.

### CRPD prevalence

CRPD is another extremely rare condition only seen in a single patient.

## Discussion

### Definitions, classifications

The dystonia definition used here is the current one. It is based on the clinical symptomatology of the dystonic muscle hyperactivity. Unlike in previous studies, it includes psychogenic dystonia, rather than separating it as pseudo-dystonia. Other diagnostic systems previously used, such as the International Statistical Classification of Diseases and Related Health Problems (ICD), Diagnostic and the Statistical Manual of Mental Disorders (DSM) do not reflect our current understanding of dystonia.

The dystonia classification used in our study is currently the most frequently used one. It tries to reflect clinical needs as it is mainly based on dystonia localisation. It also reflects sectorial aspects of the health care system, such as treatment in different medical specialties. However, in our study we extended the classification system to include pathophysiology, such as task-specificity (WC, MD). Symptomatic aetiology as in diffuse brain damage (perinatal brain damage/infantile cerebral paresis, hypoxic brain damage) was excluded in our study, as the clinical symptomatology is usually dominated by non-dystonic features and so is its treatment. Localised brain damage, however, was included, although only one HD patient with focal contralateral basal ganglia lesion occurring in temporal relationship was identified. TRD as another symptomatic aetiology was included, as it is a rather common dystonia form and as its iatrogenic origin deserves special medical attention. In addition, its clinical features are specific and require special therapeutic approaches. Altogether, our classification is similar to a recently suggested one [1]. It was not the purpose of this study to suggest a novel classification system or to decide on the appropriateness of existing classification system. The data reported here can easily be fitted in any of the existing classification systems to allow comparisons.

### Design

The gold standard design to determine the prevalence of a disease in a population would be to examine this entire population by an expert. This approach is called door-to-door survey. However, such an approach is not feasible in dystonia for several reasons: the presumed dystonia prevalence is so low, that extremely large populations would have to be screened to keep statistical errors reasonably low. In addition, the severity spectrum of dystonia is extremely large, so that such an approach would retrieve large numbers of persons, where dystonia might be a subtle finding rather than a complaint without a consequence for the patient or the health care system. This is a frequent finding, when we examine family members of our dystonia patients, or when we examine dystonia patients in all of their body parts. We, therefore, decided to use a pragmatic approach by focussing our study on the patient and its therapy and the health care system requirements (Table 3). We chose to set the entry level into our study to level 5, where the patient seeks treatment and/or the physician recommends it. With this modified service-based approach, we are confident to best serve the patients' interest in recognition and awareness and the health care system's requirements for planning resource allocation. Research interests, such as describing the full spectrum of dystonia severities, would have required different designs.

**Table 3:** Levels of dystonia severity

Level	Description
1	Dystonia is noticed by a specialist only
2	Dystonia is noticed by non-specialist observers, not by the patient
3	Dystonia is noticed by the patient
4	Patient seeks diagnosis, not treatment
5	Patient seeks treatment and/or physician suggests treatment
6	Patient receives treatment

The reference area used in our study had the advantage, that barrier-free dystonia treatment has been available for many years. This should have increased dystonia awareness and should have reduced unreported cases. Both issues have been major problems in previous studies.

Dystonia treatment provided by few interconnected treatment centres in the reference area may look as a methodological disadvantage. However, we believe it increased data homogeneity and, thus, actually increased the study quality.

### Previous studies

So far, there have been some 25 studies published on the epidemiology of dystonia. For review, see [7, 11, 12]. One of these studies provides a meta-analytical comparison [12]. As the design of the original studies varies enormously, so do their results. Table 4 shows design features influencing the results of epidemiological studies. Meta-analytical data [12] combining these vastly different study designs suggest the figures shown in Table 5.

**Table 4:** List of design details different in epidemiological studies

Design feature	Examples
Dystonia definition	DMRF definition
	ICD definition
	DSM definition
Dystonia classification	Segmental dystonia
	Multifocal dystonia
	Generalised dystonia
	Psychogenic dystonia
	Dystonic tremor
Diagnostic criteria	Definition of dystonic tremor
	Definition of psychogenic dystonia
	Definition of oromandibular dystonia/bruxism
Diagnostic quality	Media campaign and self-reporting
	Non-specialists
	Movement disorders specialists
	Multiple independent examiners
Dystonia severity	No relevance threshold (door-to-door survey)
	Treatment threshold (service based designs)
Time and place of data acquisition	Since 1970
	Public and medical awareness
	Availability of classification systems
	Availability of treatment options
	Availability of reliable data bases
Sample size of dystonia population	1–879 [3]
Sample size of reference population	707–5.8mio [12]
Type of reference population	Ethnic composition
Type of data base	Central registries
	Local data base in movement disorders centre
Health care system structure	Centralised system
	De-centralised system

**Table 5:** Comparison of meta-analytical data provided by [12] with our data

Dystonia form	Prevalence [*n*/1mio]		Corrections to match Steeves et al.	Ratio [Our prevalence/prevalence of Steeves et al.]
	Steeves et al.	Our data		
All		601.1	total	
Primary/isolated dystonia	164.3	527.0	total minus: TRD, CRPS, HD, LD, PSY	3.2
Focal and segmental dystonia	153.6	500.4	total minus: TRD, CRPS, HD, LD, PSY, GD	3.3
Cervical dystonia	49.8	251.1	CD	5.0
Blepharospasm	42.4	87.5	BS	1.8
Limb dystonia	12.4	15.2	AD	1.2
Writer's cramp	16.5	55.2	WC	3.3
Oromandibular dyst	5.2	13.3	OMD	2.5
Laryngeal dystonia	15.4	24.7	SD	1.6
Generalised dystonia	4.4	26.6	GD	6.0

### Overall prevalence

The prevalence of all patients with dystonia in our study is 601.1/1mio. This includes a prevalence of 47.5/1mio for patients with non-focal dystonia (SGD = 20.9/1mio, GD = 26.6/1mio), a prevalence of 28.5/1mio for patients with psychogenic dystonia and a prevalence of 44.6/1mio for patients with symptomatic dystonia (TRD = 38.0/1mio, HD = 1.9/1mio, CRPS = 1.9/1mio), LD = 3.8/1mio). It also includes a prevalence of 32.3/1mio for patients with MD which is often not included in previous studies.

Three previous studies will now be discussed in detail, as they are similar to our study. A service-based study from Ireland [13] includes CD, BS, focal hand dystonia, SD, MD and OMD, but excludes PSY, SGD, GD, TRD, HD and CRPS. It reports an overall prevalence of 178/1mio. Our matched overall prevalence for these patient groups would be 464.1/1mio indicating a prevalence 2.6 times higher than previously thought. When PSY, non-focal and symptomatic dystonia (TRD, HD, CRPS) are included, our total prevalence is 3.4 times higher than previously thought. 17.2% of the Irish patients suffered from BS, which was thought to be so exceptionally low, that the authors attributed this to Ireland's reduced sunlight intensity [13]. Comparing this to our BS prevalence of 87.5/1mio with similar sunlight intensity as in Ireland, makes this explanation unlikely.

Another very recent register study from Finland [11] includes CD, BS, WC + AD, SD, OMD, LD, focal axial, SGD, multifocal dystonia and GD and found a total dystonia prevalence of 405/1mio. Matching our overall prevalence data to this study design would generate a prevalence of 498.3–1.2-fold more than previously reported.

The third study is a meta-analytical study [12]. It is compared to our results in Table 5. For primary dystonia, our prevalence is 3.3 times higher than previously thought. When all forms of dystonia including PSY, GD, TRD and other symptomatic dystonias are considered, our dystonia prevalence is 3.7 times higher.

In the following, we will comment on the epidemiology of individual dystonia forms.

**CD**: It may present with tonic, clonic and tremulous elements. Its pathognomonic feature is the geste antagoniste. The best know form of CD is the tonic one. Tremulous CD is the least known one. CD may, therefore, often be misdiagnosed as ET or Parkinson’s disease. Properly diagnosing tremulous dystonia becomes especially challenging, when tonic elements are missing. Therefore, we belief that CD, especially in its tremulous form, is substantially underdiagnosed. Tics are another differential diagnosis of CD. In addition, CD may be misinterpreted as nervousness or some other form of psychological instability. As in most other epidemiological studies, CD is the most common form of dystonia.

**BS**: It is the second most common form of dystonia. If it additionally involves perioral or mandibular muscles, the term Meige syndrome is used. Facial motor tics and hemifacial spasms are differential diagnoses. Psychogenic tics are extremely rare. Especially, when BS comes together with apraxia of eyelid opening, which may be the case in 30–50% of BS patients, BS may be misinterpreted as myasthenia gravis. In ophthalmology, BS is sometimes misdiagnosed as dry eye syndrome. BS may still be underdiagnosed.

**WC**: It is the third most common dystonia. It is characterised by its task specific occurrence, at least initially. Not infrequently, it is misdiagnosed as essential tremor, especially when it occurs in a clonic or tremulous form. Its severity covers a wide range. Often, it is not recognised by the patient, especially as neat handwriting these days becomes less and less of a necessity. In other patients with mild WC, diagnosis may be requested, but not therapy. These patients were not included in our study. Our standard treatment for WC is BT therapy. In mild cases and in cases with predominant finger involvement anticholinergic treatment may be tried first. Shifting writing to the non-dominant hand is also recommended to all of our patients. All of these patients were included in our study, as they requested and received therapy. WC is a relative frequent additional sign in patients with CD. In most of these cases, no treatment is requested so that these patients were not classified as having segmental dystonia. Overall, the high percentage of patients with mild forms of WC may contribute to considerable underdiagnosing of WC. BT therapy in WC has a low acceptance rate. This reflects problematic efficacy and frequent adverse effects of BT therapy. With increased experience over time, our results increased considerably, so that willingness to undergo a recommended BT therapy also increased. Regular use of ultrasound guidance further increased results and therapy willingness.

**MD**: It is another task-specific dystonia. It is a common problem amongst professional musicians affecting probably around 1% of them [2]. MD prevalence of 32.3/1mio in our study is much higher than the 5.11/1mio MD prevalence previously calculated [13]. Our figure most likely reflects a special situation, as the University of Music, Drama and Media and four professional orchestras and Germany’s second largest conservatory attract large numbers of musicians to the reference area. Whether Irish data on MD in the normal population are representative for other populations, need to be studied, as the proportion of musicians in the Irish population may be higher than elsewhere. The male preponderance seen in our study, was also reported previously [2, 13],

**TRD:** It is the fifth most frequent dystonia. TRD describes the aetiology of a dystonia rather than its localisation in the body. In TRD exposure to neuroleptics and other dopamine receptor blocking agents is the cause of dystonia. TRD usually consists of mixed dystonia with tonic, clonic and tremulous elements. It is predominantly localised in oromandibulolingual muscles. Periocular and axial muscles are also frequently affected. We feel, that over the last 20 or 30 years with the development of atypical and second generation neuroleptics the overall prevalence of TRD has come down. In addition, we feel that their presentation has changed with rapid lingual movements occurring less frequently. Low acceptance of BT therapy in TRD may be caused by difficult to treat dystonia manifestations and psychiatric co-morbidity.

**PSY**: It is the sixth most frequent dystonia. It describes dystonia of psychogenic origin. It is often localised in the arms and the neck. In the past, it was often termed pseudo-dystonia, but according to our current understanding, it is considered true dystonia. It is rarely if ever included in epidemiological studies on dystonia. Hints towards PSY include rapid onset, intermittent course, symptomatology mixing different movement disorders, demonstrative character, distractibility, changes in various functional contexts and co-existence of non-motor signs and multiple complaints. Psychiatric co-morbidity is more frequent than in the normal population. It may be difficult to diagnose for the non-expert. As we know very little about the time course of the condition, the real prevalence of PSY is difficult to estimate as spontaneous remissions may occur before the patient is connected to an expert.

**GD**: It is the seventh most frequent dystonia. As outlined above, GD describes the localisation of dystonia. It does not necessarily describe the severity of dystonia. In the past, this distinction was not always consequently followed, so that wide-spread, but less severe dystonia may not have been named GD. Treatment includes deep brain stimulation, BT therapy (alone or in combination with deep brain stimulation) and drug therapy.

**SD:** It is the eighth most common form of dystonia. In mild forms, it may produce hoarseness of voice only, sometimes leading to the misdiagnosis of chronic infection. In more severe cases, SD may be the cause of major disability. SD patients in this study were either primarily seen by TC-DD and then sent to TC-MP for confirmation of diagnosis and treatment, or they were primarily seen and treated by MP and then sent to DD for a comprehensive dystonia work-up. SD is a relatively common additional manifestation of CD and other, more wide-spread forms of dystonia.

**SGD:** It is the ninth most common form of dystonia. It usually consists of CD and arm involvement. Mild arm involvement is relatively common. Our data only include arm involvement if therapy is required and/or requested.

**AD:** It is the tenth most common form of dystonia. Isolated AD is rare. It may have developed from task- specific dystonia, when this has become continuously occurring.

**OMD:** It is the eleventh most common form of dystonia. According to our definitions, OMD excluded all patients with oromandibular dystonia caused by neuroleptics exposure. In four of the seven patients with OMD seen here, OMD was nocturnal, so that the term bruxism may be used. None of those patients had a positive family history; one had additional signs of CD. Two of the three patients with continuous OMD had a positive family history. We believe that nocturnal OMD, conventionally called bruxism, may in fact be an attenuated form of OMD. If that would be the case, the overall incidence of dystonia would be considerably higher than currently believed.

**LD:** It is an extremely rare form of dystonia. In early onset dystonia it is usually a transient stage in the development of DYT1-positive generalised dystonia. Our two LD patients suffered from iatrogenic dopa-induced leg dystonia.

**HD:** is also a very rare condition caused by contralateral basal ganglia damage, as it was the case in our patient.

**CRPD:** It is another very rare form of dystonia. Chronic pain syndromes including CRPS seem to have the potential to produce movement disorders, such as painful leg and moving toe syndrome [6].

## Conclusions

Prevalence figures of dystonia are heavily influenced by numerous methodological aspects including dystonia definition, dystonia classification, diagnostic criteria, diagnostic quality, time and place of data acquisition, sample sizes of dystonia population and reference population, type of reference population, type of data base and structure of the health care system. We chose design parameters to provide data relevant to the patient's therapy and the health care system requirements.

For this, we used the current dystonia definition, the current dystonia classification without excluding any dystonia group, using dystonia experts for diagnostic quality and chose the need for therapy as the severity parameter. The reference population was a large unbiased central European one with a well-established comprehensive dystonia infrastructure, barrier free treatment and a high degree of dystonia awareness all contributing to high dystonia retrieval rates.

Our adjusted overall dystonia prevalence is more than double the prevalence of the latest comparable study. When PSY and symptomatic dystonia such as TRD, HD and CRPD are included, our prevalence more than triples the previous one.

As with all service-based prevalence studies a certain unidentified proportion of dystonia patients remains. How big this proportion is, remains unclear. Further retrieval rate studies will have to address this.

Prevalence figure provided in this study may be used to plan resource allocation for comprehensive dystonia therapy.
